# The glucotoxicity protecting effect of honokiol in human hepatocytes *via* directly activating AMPK

**DOI:** 10.3389/fnut.2022.1043009

**Published:** 2022-11-18

**Authors:** Hui Liu, Wu Luo, Jiazheng Liu, Xincong Kang, Jianming Yan, Tingting Zhang, Lan Yang, Lu Shen, Dongbo Liu

**Affiliations:** ^1^Horticulture College, Hunan Agricultural University, Changsha, Hunan, China; ^2^State Key Laboratory of Subhealth Intervention Technology, Changsha, Hunan, China; ^3^Hunan Provincial Engineering Research Center of Medical Nutrition Intervention Technology for Metabolic Diseases, Changsha, Hunan, China; ^4^State Key Laboratory of Quality Research in Chinese Medicine, Macau University of Science and Technology, Macau, Macau SAR, China; ^5^National Research Center of Engineering Technology for Utilization of Botanical Functional Ingredients, Hunan Agricultural University, Changsha, Hunan, China

**Keywords:** honokiol, glucotoxicity, glucose metabolism disorder, oxidative stress, type 2 diabetes, AMPK

## Abstract

**Introduction:**

Sustained hyperglycemia causes glucotoxicity, which has been regarded as a contributor to hepatocyte damage in type 2 diabetes (T2D) and its metabolic comorbidities. Honokiol is a natural biphenolic component derived from the dietary supplement *Magnolia officinalis* extract. This study aimed to investigate the effects of honokiol on glucose metabolism disorders and oxidative stress in hepatocytes and the underlying mechanisms.

**Methods:**

HepG2 cells were treated with glucosamines (18 mM) to induce glucotoxicity as a diabetic complication model *in vitro*.

**Results and discussion:**

Honokiol significantly increased glucose consumption, elevated 2-NBDG uptake, and promoted GLUT2 translocation to the plasma membrane in glucosamine-treated HepG2 cells, indicating that honokiol ameliorates glucose metabolism disorders. Furthermore, glucosamine-induced ROS accumulation and loss of mitochondrial membrane potential were markedly reduced by honokiol, suggesting that honokiol alleviated glucotoxicity-induced oxidative stress. These effects were largely abolished by compound C, an AMPK inhibitor, suggesting an AMPK activation-dependent manner of honokiol function in promoting glucose metabolism and mitigating oxidative stress. Molecular docking results revealed that honokiol could interact with the amino acid residues (His151, Arg152, Lys243, Arg70, Lys170, and His298) in the active site of AMPK. These findings provide new insights into the antidiabetic effect of honokiol, which may be a promising agent for the prevention and treatment of T2D and associated metabolic comorbidities.

## Introduction

The incidence and prevalence of type 2 diabetes (T2D) are on the rise worldwide and have reached alarming levels. It is estimated to increase from 536.6 million in 2021 to 783.2 million by 2045 (1). Microvascular (retinopathy, nephropathy, and neuropathy) and macrovascular (atherosclerosis) complications associated with diabetes are the major causes of disability and mortality among patients with T2D (2, 3). Sustained hyperglycemia in T2D impairs insulin-stimulated glucose uptake and utilization by peripheral tissues, including the liver, which gives rise to insulin resistance (IR) (4, 5). In the liver, IR impedes the normal glucose metabolism process, which elevates hepatic endogenous glucose production through increased gluconeogenesis and decreased glycogen synthesis (6). Accumulation of excess glucose in hepatocytes induces glucotoxicity (7). In a diabetic liver, glucotoxicity exacerbates oxidative stress, eliciting glucose metabolism disorders (8, 9).

The nutrient-sensing hexosamine biosynthetic pathway (HBP), a glucose metabolic pathway branching off from main glycolysis, mediates glucotoxicity and is strongly associated with diabetic complications (10–12). As a metabolite of HBP, glucosamine increases HBP flux and promotes IR by inhibiting insulin-stimulated glycogen synthesis and glucose uptake in hepatocytes (13, 14). It also impairs glucose transporter 2 (GLUT-2) translocation from an intracellular pool to the plasma membrane, reducing glucose uptake in hepatocytes (15). In addition, glucosamine-induced glucotoxicity impairs the mitochondrial function in hepatocytes, increases ROS production, and causes oxidative stress (16). Hence, it is of great significance to develop natural plant extracts that improve glucose metabolism disorders and alleviate oxidative stress in hepatocytes to prevent the occurrence and development of diabetes and its complications.

AMP-activated protein kinase (AMPK) is a serine/threonine protein kinase that regulates cellular energy homeostasis and plays an essential role in hepatic metabolism (17, 18). Liver AMPK controls glucose homeostasis mainly by suppressing hepatic glucose production and decreasing the expression of genes involved in hepatic gluconeogenetic genes, which protects against hepatic IR (19, 20). Under fasting conditions, AMPK activation enhances energy metabolism by upregulating mitochondrial function in the liver to respond to energy deficiency (21). Conversely, excess nutrients suppress AMPK phosphorylation in the liver and hepatocytes, which triggers increased HBP flux (22). In addition, activation of AMPK alleviates oxidative stress by reducing fatty acid synthesis, promoting NADPH synthesis, and limiting its consumption for maintaining cellular redox and metabolic homeostasis (23, 24). Collectively, AMPK is regarded as a potential therapeutic target against hepatic glucose metabolism disorders and oxidative stress in T2D.

Natural products from plants are excellent sources of therapeutic agents (25). *Magnolia officinalis*, a Chinese medicinal herb, includes two major species, namely, *M. officinalis* Rehder & E.H. Wilson and *M. officinalis* var. *biloba* Rehder & E.H. Wilson (26). Recently, *M. officinalis* extract has been used for dietary supplements and medicinal purposes worldwide (27–29). It contains various nutrients and phytochemical components, such as lignans, phenylethanoid glycosides, phenolic glycosides, alkaloids, steroids, and essential oils, which enable it to display anti-oxidative, anti-inflammatory, and anxiolytic effects (30). Phenylethanoid glycosides isolated from *M. officinalis* var. *biloba* fruits display free radical scavenging activities (28). Magnolol and its isomer honokiol, two lignan compounds, are major bioactive ingredients of *M. officinalis* (31). Magnolol exhibits multiple beneficial effects on T2D and its complications *via* improving glucose homeostasis, promoting lipid metabolism, and reducing oxidative stress and inflammation (32). Honokiol, an isolated dietary biphenolic natural product from the bark, leaves, and root of *M. officinalis*, demonstrated pleiotropic bioactivities such as anti-oxidation, anti-inflammation, hepatoprotection, anti-obesity, and antidiabetes (33–35). However, no previous studies have reported the glucotoxicity-protecting effect of honokiol in hepatocytes. Notably, some studies have reported that honokiol can activate AMPK phosphorylation, improving hepatocyte lipid metabolism. However, whether honokiol can directly bind to AMPK does not have computational molecular docking analyses. It is still unclear if honokiol ameliorates glucotoxicity-induced glucose metabolism disorders and oxidative stress is AMPK-dependent (36, 37).

In this study, molecular docking was performed to investigate whether honokiol could directly bind to AMPK active sites. Glucosamine was used as an *in vitro* model of diabetic complications to assess the antidiabetic activity of honokiol. We examined changes in glucose consumption, 2-NBDG uptake, GLUT2 translocation, production of ROS, and mitochondrial membrane potential in HepG2 cells treated with glucosamine with or without honokiol. Application of compound C, a selective AMPK inhibitor, estimated whether these beneficial effects of honokiol were AMPK-dependent. This study will provide new insights into the antidiabetic potential of honokiol.

## Materials and methods

### Materials and reagents

Honokiol was purchased from Shanghai Yuanye Bio-Technology Co., Ltd. (Shanghai, China). Compound C was purchased from Selleck (Shanghai, China). Dulbecco's Modified Eagle's Medium (DMEM), Dulbecco's Phosphate Buffered Saline 1X (DPBS, #14190250), trypsin/EDTA, penicillin, and streptomycin were purchased from Gibco (Grand Island, NY). Fetal bovine serum (FBS) was purchased from Biological Industries (Beit-Haemek, Israel). Dimethyl sulfoxide (DMSO) and rosiglitazone were purchased from Sigma-Aldrich (St. Louis, MO, USA). Glucosamine, Insulin, Cell Counting Kit-8 (CCK-8) Kits, RIPA Lysis Buffer, Membrane and Cytosol Protein Extraction Kit, Bicinchoninic Acid (BCA) Protein Assay Kit, Hoechst 33342 Staining Solution for Live Cells, and Mitochondrial Membrane Potential Assay Kit with JC-1 were purchased from Beyotime Biotechnology (Shanghai, China). Glucose Assay Kit was purchased from Nanjing Jiancheng Bioengineering Institute (Nanjing, China). 2-NBDG (2-(N-(7-nitrobenz-2-oxa-1,3-diazol-4-yl) amino)-2-deoxyglucose) was obtained from Invitrogen (Carlsbad, CA, USA). The primary antibodies against total AMPK, phospho-AMPK (Thr172) and GAPDH were purchased from Cell Signaling Technology (Beverly, USA). Primary antibody against glucose transporter type 2 (GLUT2) was purchased from Proteintech Group, Inc. (Wuhan, China). Anti-Na^+^/K^+^-ATPase α-1 was purchased from Jingjie PTM BioLab Co. Ltd. (Hangzhou, China). The human hepatic HepG2 cell lines were originally obtained from the Cell Bank of the Shanghai Institute of Biochemistry and Cell Biology, Chinese Academy of Sciences (Shanghai, China).

### HepG2 cell culture and viability assay

Human hepatic HepG2 cells were cultured in DMEM/high glucose supplement with 10% FBS, penicillin G (100 U/ml), and streptomycin (100 μg/ml) at 37°C in a humidified atmosphere with 5% CO_2_. The CCK-8 assay was used to evaluate the viability of HepG2 cells according to the manufacturer's instructions. Briefly, HepG2 cells were seeded in 96-well plates. After overnight incubation, the cells were treated with honokiol (3.125–800 μM) for 18 h or 24 h. Then, 10 μl of CCK-8 was added to each well, and the cells were incubated under conditions of 37°C and 5% CO_2_ for 2 h. The absorbance was measured at 450 nm using the Tecan Spark multimode microplate reader (Tecan, Männedorf, Switzerland). The following formula was used to calculate the cell viability: cell viability (%) = [A (treatment group) – A (blank)]/[A (control) – A (blank)] × 100%.

### Diabetic complication model *in vitro* and drug treatment

The diabetic complication model was induced by glucosamine, as previously described (13). Briefly, HepG2 cells were cultured in high-glucose DMEM supplemented with 10% FBS. The medium was changed after reaching 75% confluence. HepG2 cells were treated with 18 mM glucosamine for 18 h in the serum-free DMEM medium with low glucose to induce glucotoxicity. Before drug treatment, the cells were starved for 4 h in a serum-free DMEM medium with low-glucose levels. HepG2 cells were then cultured with 18 mM glucosamine and co-treated with honokiol or rosiglitazone (positive drug control) for 18 h. The control group was administered the same amount of DPBS. To determine whether honokiol regulates phosphorylation of AMPK, HepG2 cells were pretreated with the AMPK inhibitor compound C (5 μM) for 30 min, followed by treatment with 18 mM glucosamine and co-treated with honokiol for 18 h.

### Glucose consumption assay

HepG2 cells were treated with or without 18 mM glucosamine and co-treated with or without honokiol in 96-well plates for 18 h. The glucose content in the supernatant of each well was detected at 505 nm using a glucose assay kit (glucose oxidase method) according to the manufacturer's instructions. The glucose consumption in cell supernatant was calculated by subtracting the measured glucose concentration in the medium from the glucose concentration of the original DMEM media. Then, glucose consumption was normalized to the cell number using the CCK-8 assay in each well.

### Glucose uptake assay

Glucose uptake was measured using the fluorescently labeled deoxyglucose analog, 2-NBDG, as described previously (38). Briefly, HepG2 cells were seeded into a 12-well plate and were maintained in a serum-free medium for 4 h. The test compound was added to each cell sample. Following treatments for 18 h, insulin (100 nM) was administered for 30 min. Next, HepG2 cells were treated with 200 μM 2-NBDG for 30 min incubation at 37°C and then washed 3 times with PBS. 2-NBDG-treated HepG2 cells were transferred into a black 96-well plate. The fluorescence was measured using the Tecan Spark multimode microplate reader (Tecan, Männedorf, Switzerland), set at an excitation wavelength of 488 nm and an emission wavelength of 542 nm. For 2-NBDG visualization analyses, HepG2 cells were cultured in a chamber slide. The cell nuclei were stained with Hoechst 33342 staining solution for live cells for 10 min. The image was taken using a fluorescent microscope (Leica DM4000, Germany). The mean fluorescence intensity was quantified using the StrataQuest software module (TissueGnostic, Vienna, Austria). The fluorescence intensity was normalized to the cell number with Hoechst 33342. The 2-NBDG-uptake of cells was expressed as a percentage of control cells.

### Reactive oxygen species (ROS) assays

Intracellular ROS levels were generally measured using a ROS assay kit following the manufacturer's instructions. HepG2 cells were seeded in a 12-well plate and were serum-starved for 4 h. After appropriate treatment, HepG2 cells were washed 3 times with PBS and incubated with 10 μM DCFH-DA for 30 min at 37°C. DCFH-DA can freely diffuse through the cell membrane and be hydrolyzed into DCFH by ester enzymes after entering cells. Intracellular ROS can convert DCFH to fluorescent DCF. The detection of DCF fluorescence represents the intracellular level of active oxygen. HepG2 cells were washed with PBS twice (pH 7.4), and the cell nuclei were stained with Hoechst 33342 staining solution for live cells for 10 min. DCF fluorescence images were acquired using a fluorescence microscope (Leica DM 4000, Germany). ROS levels were quantified using the StrataQuest software module (TissueGnostic, Vienna, Austria). The ROS levels in cells are expressed as a percentage of control cells.

### Mitochondrial membrane potential measurement

The mitochondrial membrane potential of HepG2 cells was determined with a Mitochondrial Membrane Potential Assay Kit with JC-1. HepG2 cells were cultured with 18 mM glucosamine and co-treated with honokiol for 18 h. HepG2 cells were incubated with JC-1 at 37°C for 30 min in the dark and then washed two times with PBS. The image was taken using a fluorescent microscope (Leica DM4000, Germany). The JC-1 red/green fluorescence intensity ratio of each sample was calculated.

### Immunofluorescence assays

Membrane translocation of GLUT2 from intracellular vesicles to the plasma membrane in HepG2 cells was observed by immunofluorescence staining. Briefly, HepG2 cells were seeded on sterile glass coverslips and were gently washed 3 times with PBS. Cells were fixed in freshly prepared 4% paraformaldehyde at room temperature for 25 min. Coverslip was rinsed with PBS 3 times for 3 min each. For 30 min, 10% goat serum was used to block at room temperature, and then the goat serum was discarded. Anti-GLUT2 antibody was added, and the cells were incubated at 37°C for 1 h protected from light. After washing, cells were incubated with the Alexa Fluor 488-conjugated Donkey anti-Goat IgG (H+L) at 37°C for 1 h protected from light. The coverslips were mounted on microscope slides and sealed with an antifade solution, and nuclei were stained with DAPI. Images were captured using a fluorescence microscope (Leica DM 4000, Germany).

### Western blot analysis

The treated HepG2 cells were washed two times with PBS (pH 7.4) and lysed in RIPA lysis buffer to detect total AMPK and phosphorylated AMPK. The membrane proteins were extracted using a Membrane and Cytosol Protein Extraction Kit, following the manufacturer's instructions. Protein concentration was measured with the BCA Kit. A 5 × SDS loading buffer was added. Protein samples were separated by SDS-PAGE and transferred to a PVDF membrane. Then the membranes were incubated with specific primary antibodies, followed by incubation with the appropriate secondary horseradish peroxidase-conjugated antibodies. Immunoblots were captured using the iBright 1500 imaging system (Thermo Fisher). The levels of protein were normalized to GAPDH expression. Quantification of immunoblotting band intensity was performed using Image J.

### Molecular docking

Molecular docking was performed using Discovery Studio 2021 (Dassault Systèmes BIOVIA, San Diego, CA), following the standard procedures (39). The co-crystal structure of AMPK with an AMPK agonist was obtained from the RSCB Protein Data Bank (PDB ID: 4ZHX). 4ZHX is a structure of AMPK complexed with its agonist (Pubchem ID: 49870838), which facilitates the precise location of AMPK protein activity sites. Honokiol was docked into the active sites on the docking pocket, which was created according to the position of the agonist of AMPK. The x, y, and z coordinates of the active pocket box center of AMPK were set to 77.525835, 13.612198, and 33.299745, respectively. The radius of the active pocket was set to 17 Å. The docking study employed CDOCKER, an effective method for docking, to conduct a semiflexible docking algorithm based on molecular dynamics simulations. CDOCKER is an accurate docking technology in Discovery Studio. The binding modes were analyzed by Discovery Studio 2021.

### Statistical analysis

All of the experiments were performed using at least three independent experiments per sample, and the data were presented as the means ± SEM. Differences among multiple groups were analyzed using one-way ANOVA, and two groups were compared by a two-tailed Student *t-*test by GraphPad Prism 8. The *p*-values of less than 0.001 were considered highly statistically significant, while *p*-values of less than 0.01 or 0.05 were considered statistically significant.

## Results

### Honokiol increased glucosamine-induced glucose consumption and promoted 2-NBDG uptake in HepG2 cells

**Figure 1:**
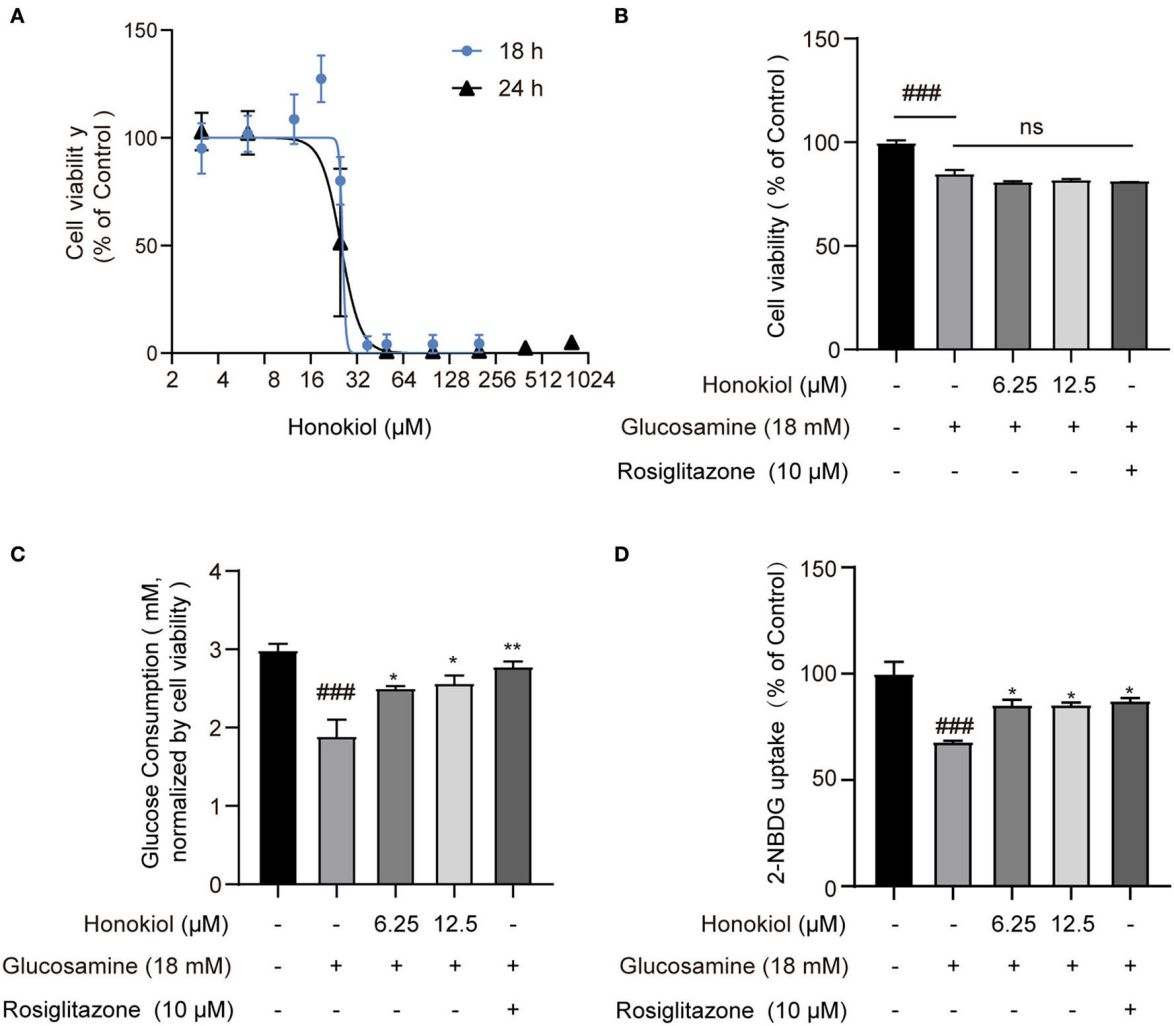
The effect of honokiol on cell viability and glucose consumption in glucosamine-induced insulin resistance in HepG2 cells. **(A)** Cell viability of HepG2 cells cultured in the presence of various concentrations of honokiol (3.12–800 μM) for 18 h or 24 h, as analyzed by CCK-8 assay; results were expressed as mean ± SEM (*n* = 6). **(B)** Relative cell viability treated with 6.25 and 12.5 μM honokiol and co-treated with/without 18 mM glucosamine. **(C)** Glucose consumption in groups treated with honokiol (6.25, 12.5 μM) and co-treated with/without glucosamine (18 mM). Rosiglitazone (10 μM) as a positive drug control. Glucose consumption was normalized by cell viability in each well. **(D)** The 2-NBDG uptake assay performed in cultured HepG2 cells. Cells in 12-well culture plates were stimulated with 100 nM insulin. After the stimulation, 2-NBDG uptake in cells was measured. Data are presented as the means ± SEM (*n* = 6). ### *p* < 0.001 compared with the control cells; *p < 0.05, **p < 0.01, *** *p* < 0.001 compared with the glucosamine-induced cells, ns indicates no significant difference compared with the glucosamine-induced cells.

Various concentrations of honokiol were tested to identify a favorable safe dosage for investigating the protective effects of honokiol on glucosamine-induced HepG2 cells. The results showed that honokiol could inhibit the proliferation of HepG2 cells in a dose-dependent manner, with 50% inhibition concentration (IC_50_) at 18 h of 25.86 μM and 24 h of 25.20 μM. Compared with untreated cells, cell viability was not obviously affected by honokiol at concentrations of 6.25 μM and 12.5 μM (Figure 1A).

The diabetic complication model *in vitro* was induced by high concentrations of glucosamine in HepG2 cells and confirmed by glucose consumption and the 2-NBDG uptake assay. HepG2 cells were treated with 18 mM glucosamine for 18 h, reducing the viability to 85.08% compared with the control group. However, the combined treatment of honokiol and glucosamine did not affect the cell viability compared with glucosamine-treated cells (Figure 1B). Glucose consumption was significantly reduced after glucosamine treatment in HepG2 cells. Honokiol and rosiglitazone (positive drug control) markedly increased glucose consumption in glucosamine-induced HepG2 cells (Figure 1C). In addition, the effect of honokiol on insulin-stimulated glucose uptake was determined by 2-NBDG. The results demonstrated that honokiol remarkably increased the glucose uptake in the glucosamine-induced HepG2 cells (Figure 1D). These results suggested that honokiol promoted glucose metabolism to alleviate glucosamine-induced glucotoxicity in HepG2 cells.

### Honokiol was predicted to bind to AMPK

**Figure 2:**
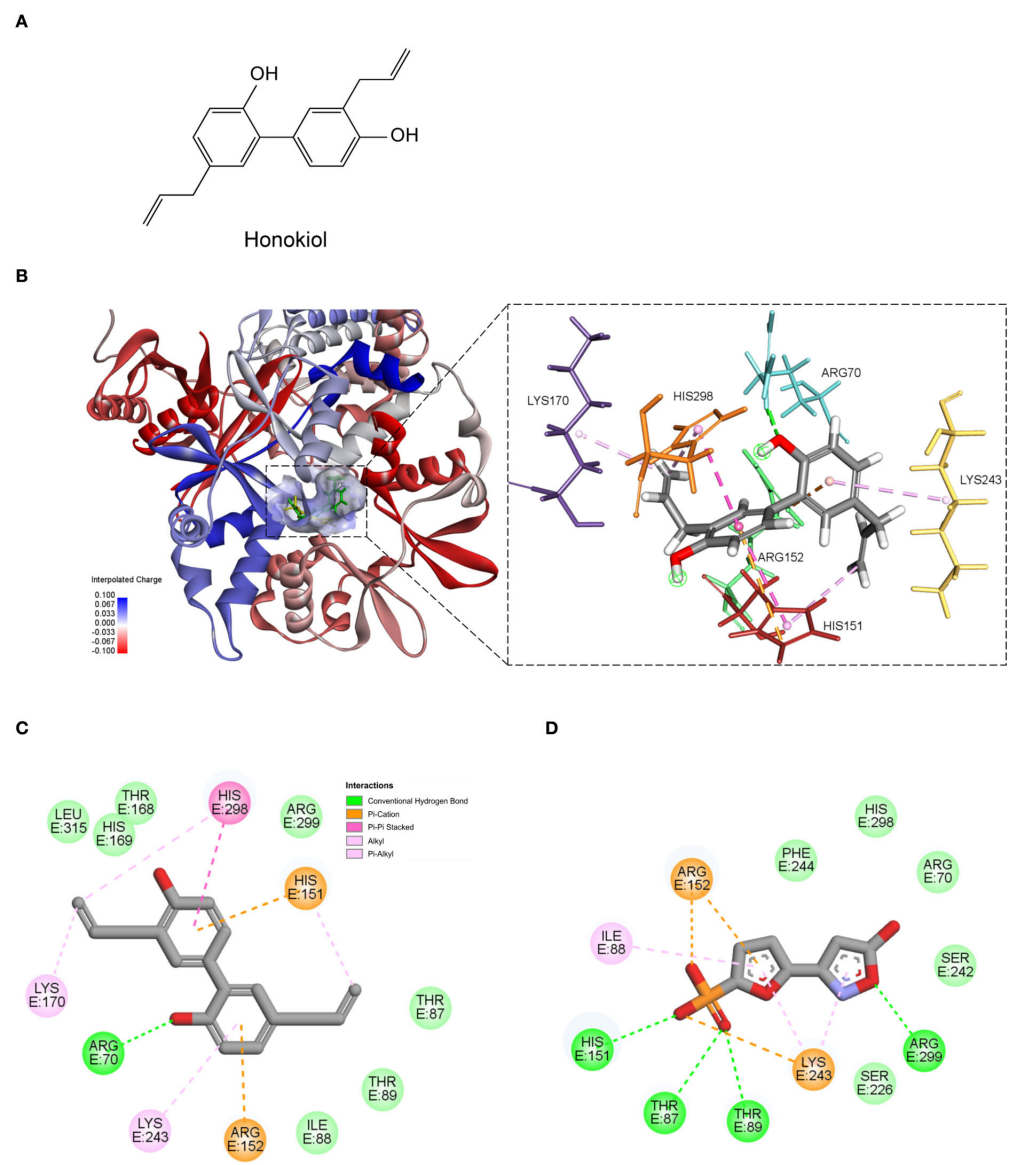
The predicted binding mode of honokiol with AMPK. **(A)** The chemical structure of honokiol; **(B)** The 3D binding models of honokiol (green); and a reported AMPK agonist (yellow) with AMPK. **(C)** The 2D binding interaction of honokiol and AMPK. **(D)** The 2D binding interaction of an AMPK agonist 5-(5-hydroxyl-isoxazol-3-yl)-furan-2-phosphonic acid, and AMPK.

Docking analysis revealed that honokiol could bind to the same active site of AMPK agonist (40). The estimated binding free energy is −14.2637 kcal/mol. Furthermore, honokiol formed interactions with residuals of His151, Arg152, and Lys243, which was in accordance with a reported AMPK agonist (Pubchem ID: 49870938). Honokiol also formed other interactions, including hydrogen bonds with Arg70 and Pi-alkyl interaction with Lys170 and His298 (Figure 2). These data suggested that honokiol is a potent activator of AMPK.

### Honokiol ameliorates glucose metabolism disorder by directly activating AMPK in glucosamine-induced HepG2 cells

**Figure 3:**
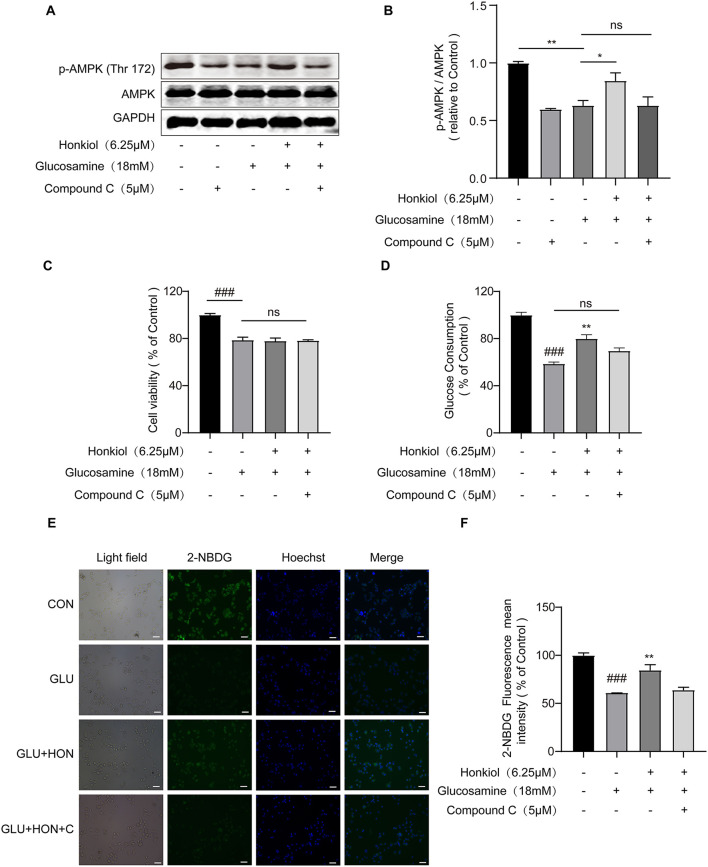
Honokiol improved insulin resistance by directly activating AMPK in glucosamine-induced HepG2 cells. HepG2 cells were pretreated with/without AMPK inhibitor compound C (5 μM) for 30 min and then glucosamine (18 mM) induced HepG2 cells co-treated with honokiol (6.25 μM) for 18 h and incubated with insulin (100 nM) for another 30 min. **(A,B)** The protein expression and relative quantitative data of p-AMPK (Thr 172) and AMPK in HepG2 cells; *n* = 3 for each group. **(C,D)** Relative cell viability and glucose consumption in groups treated with PBS, 18 mM glucosamine, 18 mM glucosamine + 6.25 μM honokiol, and 18 mM glucosamine + 6.25 μM honokiol +5 μM compound C. **(E)** The glucose uptake was then detected by 2-NDBG fluorescence probe, and the nuclei were stained with Hoechst 33342. Scale bar, 50 μm. **(F)** The mean fluorescence intensities of 2-NBDG (green) were analyzed using StrataQuest software; *n* = 3 for each group. Data are presented as the means ± SEM. ### *p* < 0.001 compared with the control cells; *p < 0.05, **p < 0.01, *** *p* < 0.001 compared with the glucosamine-induced cells, ns indicates no significant difference compared with glucosamine-induced cells. Abbreviation: CON, control; GLU, glucosamine; GLU+HON, glucosamine + honokiol; GLU+HON+C, glucosamine + honokiol+ compound C.

It was known that liver AMPK activation improved hepatic IR in hepatocytes (41, 42). Therefore, to further explore whether honokiol improved glucosamine-induced IR by activating AMPK, the HepG2 cells were pretreated with or without compound C (5 μM), a specific AMPK inhibitor, for 30 min. Then, HepG2 cells were treated with 18 mM glucosamine to induce IR and co-treated with 6.25 μM honokiol for 18 h. After treatment with compound C and glucosamine alone, the phosphorylated AMPK levels in HepG2 cells were significantly reduced. However, treatment with honokiol upregulated phosphorylation of AMPK, and compound C significantly decreased honokiol-induced AMPK activation in glucosamine-induced HepG2 cells (Figures 3A,B). Accordingly, compound C essentially abolished the beneficial effects of honokiol on glucose consumption and uptake in glucosamine-induced HepG2 cells, indicating that honokiol ameliorates glucotoxicity-induced IR by activating AMPK (Figures 3C–F).

### Honokiol promoted GLUT2 translocation from cytoplasm to the plasma membrane in an AMPK-dependent manner in glucosamine-induced HepG2 cells

**Figure 4:**
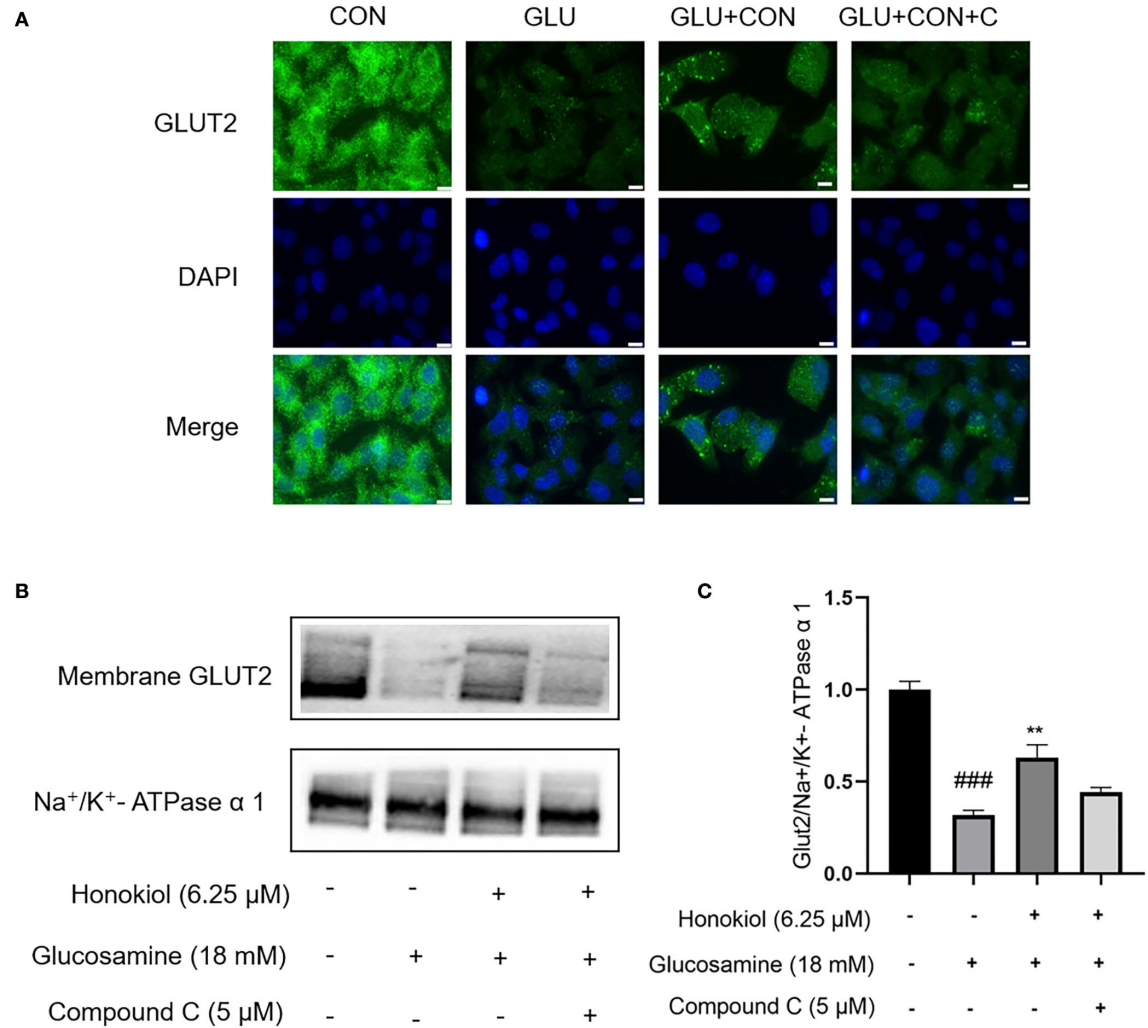
Honokiol restored glucosamine-induced impairment of GLUT2 translocation *via* activation of AMPK in HepG2 cells. HepG2 cells were pretreated with/without AMPK inhibitor compound C (5 μM) for 30 min and then were incubated with 18 mM glucosamine in the absence or presence of honokiol (6.25 μM) for 18 h and incubated with insulin (100 nM) for another 30 min. **(A)** Translocation of GLUT2 (green) from the cytoplasm to the cell membrane was assayed using immunofluorescence staining. The nuclei were stained by DAPI (blue). Scale bar, 10 μm. **(B)** Expression of membrane GLUT2 was detected by Western blot. Na^+^/K^+^-ATPase α1 was used as a loading control. **(C)** Quantification of membrane GLUT2. Data are presented as the means ± SEM. ### p < 0.001 compared with the control cells; *p < 0.05, **p < 0.01, *** *p* < 0.001 compared with the glucosamine-induced cells. Abbreviation: CON, control; GLU, glucosamine; GLU+HON, glucosamine + honokiol; GLU+HON+C, glucosamine + honokiol+ compound C.

To further investigate whether honokiol elevated glucose uptake by promoting the translocation of GLUT2 and whether the beneficial effect was related to AMPK activation, the translocation of GLUT2 from the cytoplasm to the membrane was examined by immunofluorescence assays. As shown in Figure 4, glucosamine treatment caused an apparent blockage of GLUT2 translocation in HepG2 cells compared with the control group, and honokiol significantly promoted the glucosamine-induced GLUT2 translocation, thus restoring glucose uptake in glucosamine-induced HepG2 cells. Furthermore, the honokiol-mediated GLUT2 translocation effect was largely blocked by compound C in glucosamine-induced HepG2 cells, indicating that honokiol enhanced glucosamine-induced GLUT2 translocation from the cytoplasm to the membrane by activating AMPK.

### Honokiol inhibited ROS overproduction *via* directly activating AMPK in glucosamine-induced HepG2 cells

**Figure 5:**
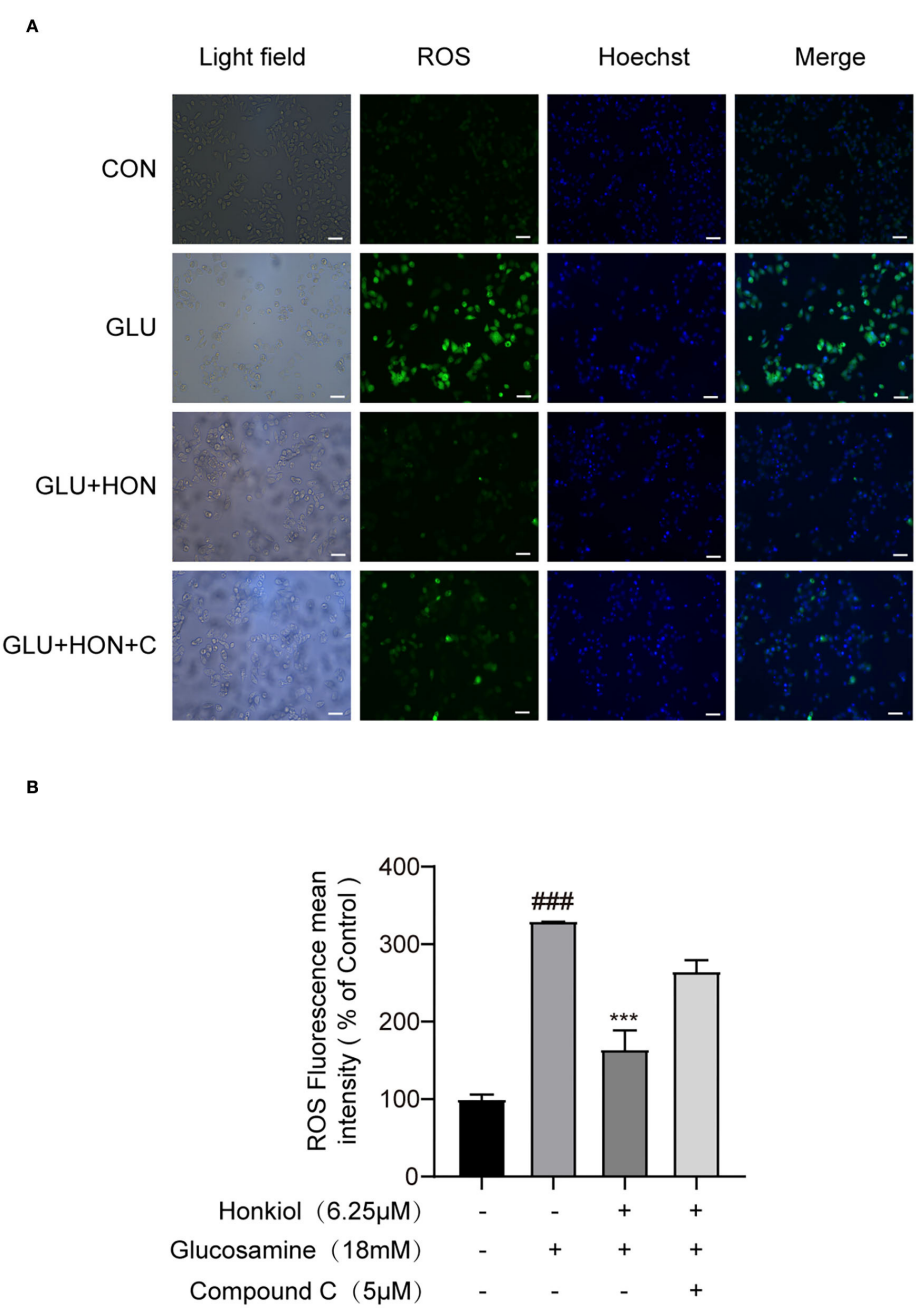
Honokiol-inhibited glucosamine-induced ROS overproduction was AMPK-dependent in HepG2 cells. **(A)** Representative images of ROS level-derived DCF green fluorescence of HepG2 cells treated with PBS, 18 mM glucosamine, 18 mM glucosamine + 6.25 μM honokiol, 18 mM glucosamine + 6.25 μM honokiol +5 μM compound C. The nuclei were stained with Hoechst 33342. Scale bar, 50 μm. **(B)** Relative mean fluorescence intensities of ROS were quantified using StrataQuest software. Data are presented as the means ± SEM (*n* = 3). ### *p* < 0.001 compared with the control cells; *p < 0.05, **p < 0.01, ***p < 0.001 compared with the glucosamine-induced cells. Abbreviation: CON, control; GLU, glucosamine; GLU+HON, glucosamine + honokiol; GLU+HON+C, glucosamine + honokiol+ compound C.

Oxidative stress was strongly associated with hepatic IR. The increased level of ROS, which led to oxidative stress, was one of the primary causes of IR (43, 44). To determine whether honokiol could eliminate excessive ROS induced by glucosamine in HepG2 cells, ROS production was determined using DCF-DA labeling, and the mean DCF fluorescence intensity was determined by image analysis. Glucosamine treatment led to increased production of ROS compared with the control group, and honokiol markedly reduced the glucosamine-induced intracellular ROS level. Treatment with compound C largely eliminated the decrease in honokiol-mediated ROS production (Figure 5).

### Honokiol prevented glucosamine-induced loss of mitochondrial membrane potential in HepG2 cells *via* directly activating AMPK

**Figure 6:**
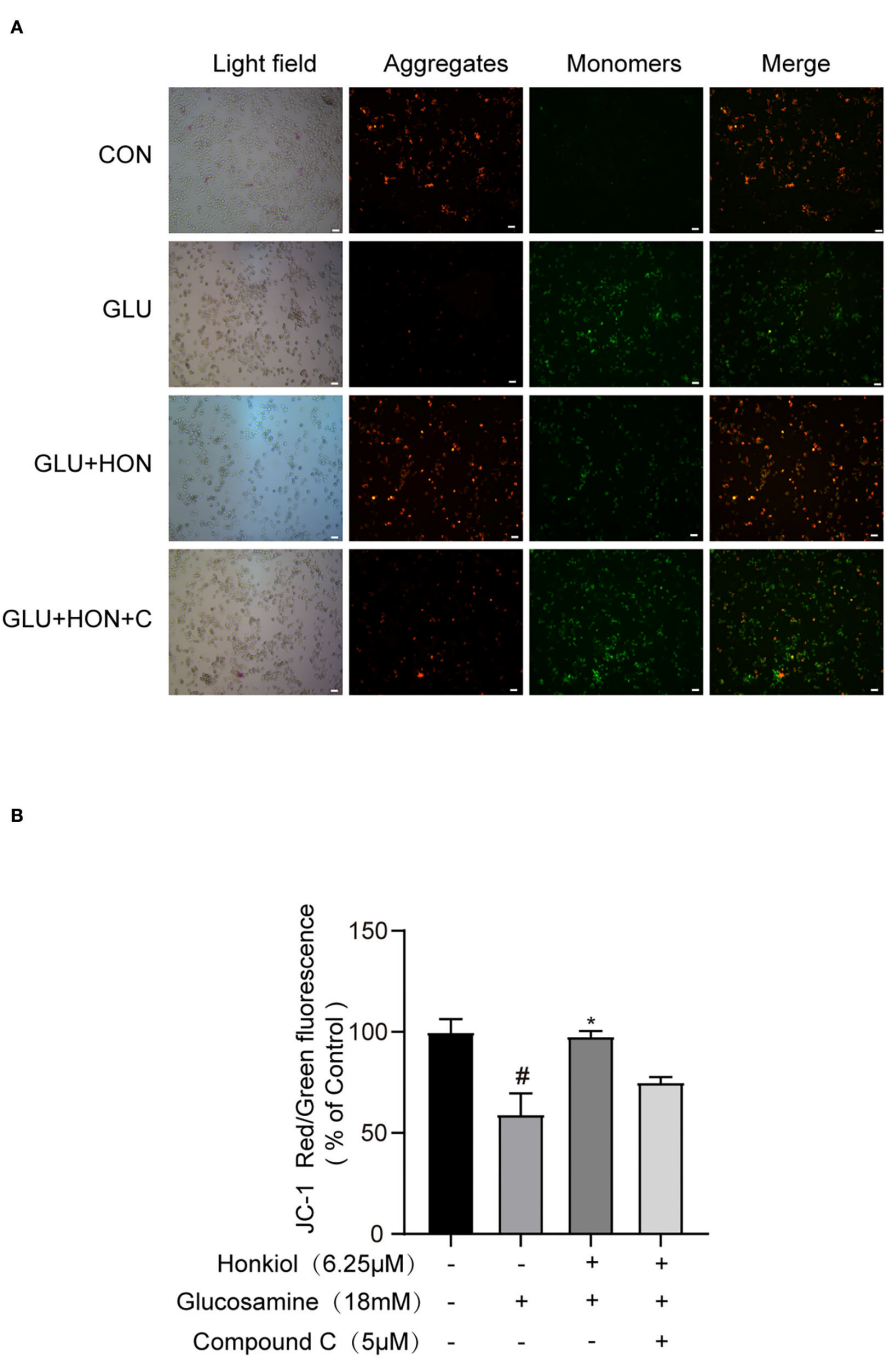
Honokiol activating AMPK to prevent glucosamine-induced loss of mitochondrial membrane potential (MMP) in HepG2 cells. The red color indicates healthy cells with high MMP, and the green color indicates low MMP. **(A)** Representative images of JC-1 derived red and green fluorescence of HepG2 cells treated with PBS, 18 mM glucosamine, 18 mM glucosamine + 6.25 μM honokiol, and 18 mM glucosamine + 6.25 μM honokiol +5 μM compound C. Scale bar, 50 μm. **(B)** Mitochondrial membrane potential calculated as the ratio of red to green JC-1 fluorescence and expressed as a percentage of control. Data are presented as the means ± SEM (*n* = 3). ### *p* < 0.001 compared with the control cells; *p < 0.05, **p < 0.01, *** *p* < 0.001 compared with the glucosamine-induced cells. Abbreviation: CON, control; GLU, glucosamine; GLU+HON, glucosamine + honokiol; GLU+HON+C, glucosamine + honokiol+ compound C.

Cellular ROS levels are closely related to mitochondrial function. Overproduction of ROS leads to the loss of mitochondrial membrane potential (45, 46). To further investigate whether honokiol could protect mitochondrial function under glucosamine-induced stress, the mitochondrial membrane potential was determined by JC-1 staining. Glucosamine alone treatment resulted in the loss of mitochondrial membrane potential compared with the control group. Honokiol co-treatment significantly reversed the loss of glucosamine-elicited mitochondrial membrane potential, indicating that honokiol had a protective effect on the disruption of mitochondrial function caused by glucosamine. The beneficial effect of honokiol on mitochondrial function was largely abrogated by inhibiting AMPK activity with AMPK inhibitor compound C, indicating that honokiol inhibited glucosamine-induced loss of mitochondrial membrane potential by activating AMPK in HepG2 cells (Figure 6). Taken together, honokiol had a positive effect on reducing oxidative stress by directly activating AMPK in glucosamine-induced HepG2 cells.

## Discussion

Fasting hyperglycemia is attributed to the increase in hepatic endogenous glucose production and gluconeogenesis caused by hepatic IR at an early stage in the natural history of T2D (47). During diabetes progression, sustained hyperglycemia induces glucotoxicity, which leads to diabetic complications (48). A limitation of diabetic complication studies is the absence of suitable *in vitro* models for rapidly screening potential antidiabetic agents. Glucosamine, an endogenous metabolite of HBP, is thought to be a promising inducer for *in vitro* models of diabetic complications studies. Persistent fasting and postprandial hyperglycemia in diabetic complications increase flux through the HBP and associated protein O-GlcNAcylation, which results in increased endogenous glucosamine that impairs insulin action and triggers oxidative stress (11, 49, 50). Exogenous glucosamine is transported into hepatocytes by GLUT-2 and further metabolized to N-acetylglucosamine (GlcNAc), the end product of HBP, which is used as a donor for protein O-GlcNAcylation (51, 52). Using *in vitro* models of glucosamine-induced hepatic IR and oxidative stress can effectively characterize *in vivo* metabolic burdens in patients with diabetic complications, which would provide some insights into the role of antidiabetic agents. In our study, exposure of HepG2 cells to a high concentration of glucosamine alone (18 mM) inhibited glucose uptake and translocation of GLUT2 from the cytoplasm to the cell membrane, which agreed with the previous study (15, 53). Meanwhile, glucosamine induces the accumulation of ROS and loss of mitochondrial membrane potential. These findings mimicked glucotoxicity-induced hepatic IR and oxidative stress in diabetic complications. Based on this model, we explored the antidiabetic effect and the mechanisms of honokiol, an active component of the dietary supplement *M. officinalis* extract.

Traditionally, *M. officinalis*, a Chinese medicinal herb, has been used for thousands of years to treat gastrointestinal disorders (abdominal distention, vomiting, diarrhea, constipation, etc.) and asthma (26, 30, 54). Recently, *M. officinalis* extract has been used as a new food ingredient and is classified as “generally regarded as safe” (GRAS) by the United States Food and Drug Administration (FDA) following independent recommendation (55, 56). For example, dietary supplements with *M. officinalis* extract possessed mild anxiolytic and anti-stress effects by reducing cortisol exposure and perceived daily stress in moderately stressed subjects (27, 57). In fact, the addition of 4% *M. officinalis* extract to chewing gum inhibited plaque formation (58).

Honokiol, as one of the primary active ingredients in *M. officinalis*, has been shown to exert antidiabetic abilities in multiple animal diabetic models. Hyperglycemia-induced oxidative stress contributes significantly to the development of diabetes and diabetic complications (15). Oxidative stress impairs mitochondrial function and is strongly associated with increased ROS and loss of mitochondrial membrane potential (59–61). There is considerable evidence supporting the antioxidant functionality of honokiol in various diseases, such as cancer, cardiovascular diseases, and neurodegenerative diseases, which has been attributed to the presence of phenolic functions in its structure (62–64). Honokiol prevented the development of diabetes and its multiple complications. As a ROS scavenger, it improved pancreatic β-cell function by attenuating oxidative stress through Nrf2/ARE pathway in diabetic rats (37) and ameliorated diabetic myocardial ischemia/reperfusion (MI/R) injury by reducing oxidative stress by activating the SIRT1-Nrf2 signaling pathway (65). Furthermore, it rescued myocardial energetic dysfunction in diabetes by activating SIRT3-mediated deacetylation of mitochondrial proteins (66) and alleviated lipid-induced hepatic IR by activating SIRT3-mediated lipophagy (37, 67). Collectedly, the antidiabetic effects of honokiol may be attributed to its anti-oxidative property and activation of the deacetylase SIRT3. In this study, we showed that honokiol ameliorated glucosamine-induced glucose metabolism disorder by reversing the reduction of glucose consumption, glucose uptake, and translocation of GLUT2 in human hepatocytes *in vitro*. A high concentration of glucosamine triggers glucotoxicity, leading to oxidative stress. Honokiol mitigated oxidative stress by reducing ROS accumulation and mitochondrial membrane potential loss in glucosamine-induced HepG2 cells. Overall, these results confirmed the protective effects of honokiol against glucotoxicity-induced glucose metabolism disorder and oxidative stress using an *in vitro* diabetic complications model.

AMPK is a vital nutrient-sensing enzyme that mediates cellular energy homeostasis, insulin sensitivity, and oxidative metabolism (68, 69). Dysregulation of AMPK activity leads to impaired glucose uptake and mitochondrial metabolism in studies of animals and humans with T2D (70, 71). High glucose inhibited AMPK phosphorylation in the liver and HepG2 hepatocytes (72, 73). Notably, an increase in protein O-GlcNAcylation is considered glucotoxicity and diabetic complications because GlcNAc is produced by HBP, a nutrient-sensing pathway (74). Increased HBP flux reduces AMPK activity, and the inhibition of O-GlcNAcylation has an antidiabetic effect through AMPK activation (75). As expected, our results in this study indicate that glucosamine treatment significantly downregulated AMPK phosphorylation in HepG2 cells.

Therapeutic interventions targeting AMPK represent one of the most effective strategies for treating T2D (41, 76). AMPK activation can improve insulin sensitivity and metabolic health (68). Metformin, a prescribed insulin-sensitizing clinic drug for T2D treatment, improves hepatic glucose metabolism for alleviating IR by activating AMPK (77, 78). However, metformin is often accompanied by side effects such as diarrhea, abdominal or stomach discomfort, and congenital disabilities in offspring (79, 80). *M. officinalis*, a Chinese medicinal herb, is widely used to treat gastrointestinal disorders, including diarrhea. As a digest-beneficial dietary supplement, its extract, combined with metformin, might represent a new and promising treatment regimen for T2D. Here, we sought to investigate the role of AMPK in the effects of honokiol on glucose metabolism disorders and oxidative stress in glucosamine-induced HepG2 cells. In our study, honokiol effectively activated AMPK in glucosamine-induced HepG2 cells. The molecular docking result showed that honokiol binds to the same active site of AMPK agonist (Pubchem ID: 49870938), suggesting that honokiol, as a potential AMPK agonist, can directly bind to AMPK. Therefore, we postulated that activation of AMPK might be involved in the protective effects of honokiol on hepatic IR and oxidative stress. To test this possibility, HepG2 cells were pretreated with an AMPK inhibitor, compound C. As expected, compound C significantly inhibited the effects of honokiol on glucose consumption, GLUT2 translocation, ROS accumulation, and damage of mitochondrial membrane potential in glucosamine-induced HepG2 cells. Our results suggest that AMPK is required for honokiol to ameliorate glucosamine-induced glucose metabolism disorders and oxidative stress in HepG2 cells.

## Conclusion

**Figure 7:**
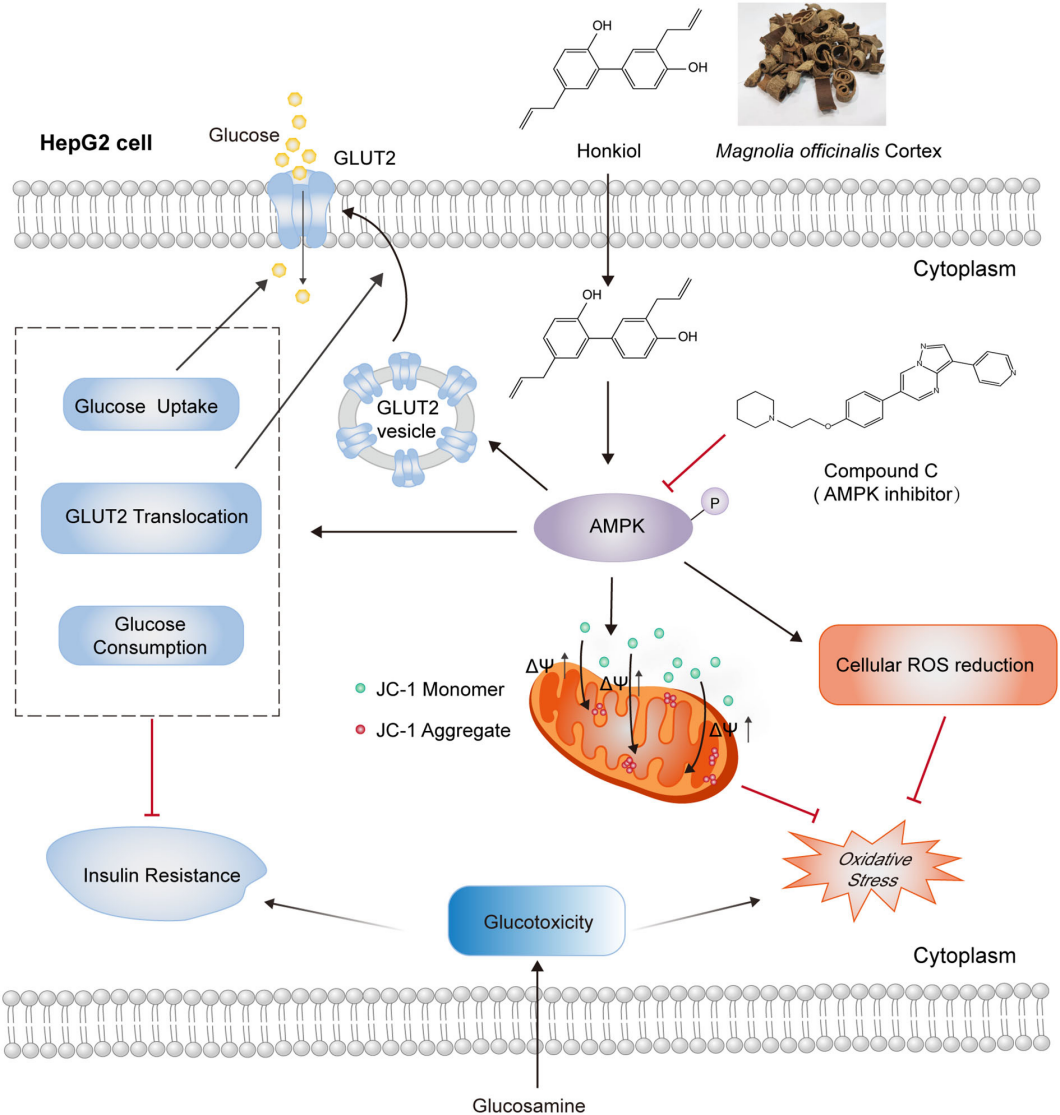
Schematic representation of AMPK-dependent antidiabetic activity of honokiol in glucosamine-induced HepG2 cells. Honokiol, an active component of the dietary supplement *Magnolia officinalis* extract, directly activates AMPK, which improves glucose metabolism disorders by increasing glucose consumption, 2-NBDG uptake, and GLUT2 translocation and alleviates oxidative stress by reducing ROS accumulation and loss of mitochondrial membrane potential in glucosamine-induced HepG2 cells. These effects of honokiol on glucotoxicity-induced glucose metabolism disorder and oxidative stress were largely abolished by AMPK inhibitor compound C.

This study provides the first experimental evidence for the glucotoxicity-protecting effect of honokiol in glucosamine-induced hepatocytes, an *in vitro* diabetic complication model. Honokiol ameliorated glucose metabolism disorder by increasing glucose consumption, insulin-stimulated 2-NBDG uptake, and translocation of GLUT2 and alleviated oxidative stress by reducing ROS accumulation and loss of mitochondrial membrane potential in glucosamine-induced HepG2 cells. Molecular docking analysis suggested that honokiol could bind to AMPK directly. Honokiol activated AMPK activity in glucosamine-induced HepG2 cells. These effects of honokiol on promoting glucose metabolism and alleviating oxidative stress were largely eliminated by AMPK inhibitor compound C (Figure 7). These findings provide new insights into the antidiabetic effect of honokiol, which may be a promising agent for preventing and treating T2D and its complications.

## Data availability statement

The raw data supporting the conclusions of this article will be made available by the authors, without undue reservation.

## Author contributions

DL: conceptualization, funding acquisition, project administration, resources, and writing—review and editing. HL and WL: data curation, investigation, and visualization. WL and XK: formal analysis. HL, WL, and JL: methodology. WL and JL: software. XK and LY: supervision. JY, TZ, and LS: validation. WL, HL, and XK: writing—original draft. All authors have read and agreed to the published version of the manuscript.

## Funding

This study was supported by the National Natural Science Foundation of China (81773850) and the Science and Technology Major Project of Hunan Province (2017SK1020).

## Conflict of interest

The authors declare that the research was conducted in the absence of any commercial or financial relationships that could be construed as a potential conflict of interest.

## Publisher's note

All claims expressed in this article are solely those of the authors and do not necessarily represent those of their affiliated organizations, or those of the publisher, the editors and the reviewers. Any product that may be evaluated in this article, or claim that may be made by its manufacturer, is not guaranteed or endorsed by the publisher.

## Abbreviations

T2D: type 2 diabetes
IR: insulin resistance
AMPK: AMP-activated protein kinase
p-AMPK: phosphorylated AMPK
2-NBDG, 2-(N-(7-nitrobenz-2-oxa-1: 3-diazol-4-yl) amino)-2-deoxyglucose
GLUT2: glucose transporter 2
GLUTs: glucose transporters
ROS: reactive oxygen species
GLU: glucosamine
HON: honokiol
DMEM: Dulbecco's Modified Eagle's Medium
DMSO: dimethyl sulfoxide
FBS: fetal bovine serum
CCK-8: cell counting kit-8
IC_50_: the half-maximal inhibitory concentration
GAPDH: glyceraldehyde-3-phosphate dehydrogenase
HBP: hexosamine biosynthetic pathway.
